# Epilepsy

**Published:** 1862-08

**Authors:** Edward Sutton Smith

**Affiliations:** No. 10 Union Square, New York


### EPILEPSY.

AFTER THE FRENCH OF HERPIN.

By EDWARD SUTTON SMITH, M.D.

Art. II. Continued from page 438.

*Interval between Attacks.*—When one does not follow step by step the succession of epileptic attacks, they will be struck by their apparent irregularity; but if a certain lapse of time be considered as comprising a great number of acces, and this time is divided into equal periods; or, which amounts to the same thing, if you will take in fractions the daily minimum of the acces during irregular periods—for instance, during such and such treatment—it will be found that the return of the attacks is conformable, at least during a certain time, to a kind of regular law that was not at first suspected.

When we examine with attention a long series of the dates of acces, and keep account of the order by paroxysms when they occur, the intervention of certain accidental attacks, or the non-arrival of the ordinary return, we shall arrive at the conclusion that, though without the regularity of intermittent fevers, the crises of epilepsy are much less abnormal in their appearance than has been generally supposed, and that they are really regular, which distinguishes them from all other nervous crises.

In incurable cases, sooner or later the return of the acces is manifested by an acceleration, which is a bad sign. This gradual acceleration, generally slow, though sometimes rapid, is the indication of the course of the disease.

I have encountered all degrees of frequence; from more than one hundred a day, down to a single attack every four or five years. The most numerous category is that where patients have had from one to six attacks a week. In hospitals, in consequence of there being no infants, monthly attacks are usually those which are reported.

Acces return and follow two distinct courses; either by isolated acces, or by paroxysms; sometimes these two kinds combine, and at other times change from one to the other. The first course is three times as frequent as the latter.

Generally, the paroxysms come on about every month, lasting from one to three days, and are composed of from one to six acces.

Accidental or very intense paroxysms sometimes cause death.

Diurnal acces are sometimes as frequent as nocturnal, if we take for a day the time that elapses between the rising and retiring of the patient.

The lighter the crises are, the more apt they will be to appear during the day.

Nocturnal attacks are generally more intense, and indicate a higher degree of the malady.

In general, the interval between the attacks grows shorter as the disease advances.

*Nature of Acces—Section First—Type of an Acces of Epilepsy.*—An individual, apparently in perfect health, without any premonition, gives a cry, and suddenly falls, deprived of intelligence. At once tonic convulsions commence at some point of the locomotive system, and ultimately extend to all the voluntary muscles. The contraction, in proportion as it is more energetic in the muscles of one side of the body than in those of the other—in the extensors than in the flexors, in the elevators than in the depressors, in the constrictors than in the dilators—induces such a variety in the fixed attitude of the members of the trunk, head, or the expression of the countenance. From this immobility of the muscles and occlusion of the glottis results, as has been well shown by Van Swieten* and M. Beau, the suspension of respiration, then all the signs of asphyxia; we will add, as a natural consequence, the accumulation of saliva in the mouth, arising from the compression of the salivary glands.

This period is called *tetanic*.

After this first division, to the tonic phenomena are added, first, partial, then general, clonic convulsions; which, according to their predominance in such or such muscles, or such and such regions, produce the most irregular movements and hideous expressions of the face.

Respiration, incompletely re-established by these movements, is performed with considerable noise; you hear the noise produced by the passage of the air through the mucus or saliva, which soon appears upon the lips in the shape of foam. This foam is sometimes reddened by the blood that arises from

* Commentaria in H. Boerhaavii Aphorismos, Parisiis, 1758, tome III., p. 398.

the biting of the tongue. The urine passes involuntarily, moistening the garments of the patient.

This the second phase, that of the *shocks.*

The *rale* and the rigidity evidently persist after the clonic convulsions, then diminish, and finally cease more or less rapidly during the third period. This latter is marked by muscular relaxation, coma, and stertorous breathing. It is the perfect image of that condition which is known by the name of *cerebral commotion.* The loss of intelligence continues; the pupils are greatly dilated; respiration is low, profound and stertorous; the face is less colored; the pulse, from having been rapid during the preceding periods, now gradually lessens.

This is the snoring period, or *comatose state.*

The patient wakes up as if from a prolonged sleep, astonished, exhausted, terribly fatigued, and with a pain in the head. Has no recollection whatever of the painful scene that has just passed. Can articulate a few words, but soon falls into a sleep that is really reparative.

This fourth period is that of *return to consciousness.*

The duration of the first period is very short—about a quarter of a minute; each of the succeeding ones is about four times as long as that which immediately precedes it.

Such is the type of a complete attack of epilepsy; such is the result according to my idea, of the numerous descriptions given by authors of the comparison of a number of facts; and finally, of my own observation. But this type, like all others, is in reality but a generalization, and we must not expect to find it exactly reproduced in the majority of epileptic cases. The different degrees of intensity, and the greater or less duration of the acces, present numerous varieties; more numerous than any physician would suppose, who had not specially studied this disease.

The study of all these varieties has never been methodically pursued, and this subject, alone all-important from a diagnostic point of view, we shall undertake, though without the pretension of calling it complete, for, to accomplish that one should live in and epileptic hospital.

*Section Second—Prodromes.*—The prodromes are divided into *remote* and *near;* in this latter category are included those sensorial or convulsive manifestations that from their nature are intimately connected with an attack properly so called.

We have encountered prodromes only in nine individuals out of thirty-five; that is, in scarcely a quarter of the cases. Those observed were of a very variable nature.

Change of character: irritability, turbulence, violence, sadness, agitation, struggle against the attack.

Vertigoes.

Headache, congestion of the head, somnolence, general fatigue.

Nervous trembling.

Skin burning; sensation of cold; chills; somtimes paleness.

Nausea.

Diarrhœa; cholic, without evacuations.

But these prodromes as signs are so far from having that value that has been attributed to them. If the patient is watched a long time, and the precursory signs noted with care, it will be found that the prodromes are often wanting, and that even their appearance is not always followed by an attack.

*Section Third—The Commencement of an Attack, and the Aura Epileptica.*—The cases in which we can follow an attack of epilepsy from its origin are very rare, particularly in private practice. Even when and acces has taken place in the presence of a physician (unless he has been forewarned) who is both attentive and placed in favorable circumstances, it is difficult to observe from its very commencement. But, happily, there are cases where the phenomena do not follow each other so rapidly; where it is possible to study the initial steps of the attack from the information furnished either by the patient or his friends.

The first attack that took place in Observation No. 2, commenced with a cramp or contraction with flexion of the two last fingers of the left hand; she then gave utterence to two or three screams, while her hand closed, her forearm bent, and her whole arm was raised in such a manner as to carry the hand towards the shoulder; the succeeding contractions were very painful; the head turned around; it seemed to the patient that the same sensation was about to pervade the whole body; consciousness was lost; and finally the young girl fell upon the floor, and had a well-marked attack. Same state of things took place in the next acces. All these details are authentic, having been given me by the girl herself, without my directing her by questions; her friends, intelligent persons, confirmed the facts.

*Observation No. 9—(Third Series.)*—Miss W., of Brooklyn. Disease first manifested itself in 1859; interval between attacks about 14 days; always commence with cramps in the left foot, which gradually extend to the trunk, till the whole body is convulsed; has had as many as eight attacks in twelve hours. Came under treatment the 16th March, 1862; has never had an attack since.

We shall find a similar case in observation No. 38. Painful cramp in the great-toe of one foot, which produced groans, and sometimes screams; the toes had a tending to turn up; the painful sensation, accompanied with a sense of fulness, gradually gained the limb, the thigh, the stomach, the chest, passed into the fingers of the hand; some of them turned, remounted to the shoulder, the side of the neck, the ears, the face, and terminated at the top of the head; then the patient lost her sight, the head turned upon its axis, or backward; consciousness vanished; the members straightened out and stiffened; the close of the attack was quite short, and marked by some shocks of the head.

As regards the fall, the duration of the *debut* always allowed this second young lady to sit down and sustain herself, otherwise she would certainly have fallen. Observe that, in this last case, the cramp sometimes commenced in the leg, the thigh, the fingers, etc., and sometimes passed from one side to the other, but always advancing from below upward on the same side. The details of this second case were as correct as those of the first. Having been myself a witness to one of these cases, I can bear witness to their minute exactness.

I shall here cite Bonet*—interesting from the double point of view of the influence of the ligature, and the preservation of intelligence during the attacks.

"Many sympathetic cases of epilepsy have been cited, dating their origin from many different parts of the body, but none have ever been published where the affection commenced in the inguinal region. I observed a case of this kind in 1656, in the person of M. Rosselet, of Neufchatel, in Switzerland. He was a man 50 years of age, red hair, a *bon vivant*, and somewhat addicted to the society of the fair sex during his youth, as he himself said. At intervals he felt in the left groin a swelling like a small bubo; from thence a crawling sensation passed slowly down the thigh to the very sole of the foot. When it had arrived there, a kind of wind or vapor (*vapor quidam quœris aura*) mounted rapidly from this point to the brain, though it only reached the left side, as could be seen from the fact that the convulsions only affected the left side of the body and face, which was deformed by frequent grimaces. The patient lost neither speech nor consciousness, though this latter was altered, the vapor also reaching the nerves of the tongue. He said hesitatingly to me, in the midst a paroxysm, '*See, see, how this terrible evil torments me!*' He experienced, at the same time, shocks in the arms and legs; this condition of things lasted

* Sepulchrum, folio 1700, t. I., sec. 12.

about five minutes." The patient, to whom Bonet proposed the application of the actual cautery at the point of departure and throughout the length of the course of *the aura*, refused in the most absolute manner. "He consented, however, to employ a ligature above or below the knee, so as to intercept the course of *the aura*. The moment that this latter commenced, and the patient felt the itching and tumefaction of the groin, he drew tightly around his leg a garter that always hung on his bed. He always succeeded in arresting the paroxysm, and gave up all other remedies. One evening, while dining with his wife and children, who had pushed the table against his bed, he felt the vapor coming on; the obstacles that prevented his taking his garter could not be removed with sufficient celerity; the vapor became all the more violent from having been so long compressed, and mounted with such fury that this eminent man perished in the paroxysm."

The following is the opinion of Esquirol on the subject: "In a great number of cases, epileptics, before losing their consciousness, feel a convulsive movement, a kind of pain; they experience a chil, a vapor (aura epileptica) on the head, the face, one of the arms, the hands, the thighs, the legs, the toes; these different feelings are propagated the whole length of the limbs, towards the head, and when this *vapor* arrives at the head the patient loses consciousness; then the convulsions are sometimes general, sometimes partial, and sometimes limited to the limb that was primarily affected."

As we advance in the seventeenth and eighteenth centuries, we find the cold vapor less and less frequently mentioned; it is replaced by spasms, cramps, convulsions, and shocks; and finally, in our time, criticism, the child of doubt, has denied the phenomena altogether.

From a close analysis of the most complete and recent facts, it seems ligitimate to conclude that the prodrome described under the name of *aura epileptica* is nothing more than the first convulsive manifestation of the attack, taking place in a part of the system more or less removed from the brain.

To give a definite *resume* of the subject—

*The functional alteration of the nervous centres, which constitutes an attack of epilepsy, usually invades those organs from the superior part of the cerebro-spinal axis to its inferior portion; but it may also commence at various points throughout the length of this system, as well as in its inferior portion. This last form is the severest of all.*

I am inclined to think that the cry, which is the second phenomenon at the commencement of an attack, *is an expression both of surprise and of the pain caused by the convulsion. The cry is met with in about half of those epileptics that have complete attacks, but it is only constant and reliable in about a quarter of the cases. In the case of children it is often replaced by tears.*

This symptom is never connected with vertigoes or incomplete acces. Generally speaking, the most violent attacks are those that are preceded by the cry or scream.

The fall is the third symptom of an attack; it is much more frequent than the cry; without, however, being constant, and may be so violent as to cause death.

When the period of cramp or partial initial convulsions is prolonged, the acces is more generally incomplete, or very brief.

One strong scream, on the contrary, indicates the commencement of a violent attack.

The more sudden the fall, generally the shorter the acces.

In a word, *the longer the debut, the less violent the crisis; the more sudden, the more intense the acces.*

No. 10 Union Square, New York.

